# Survey of electroencephalography usage and techniques for dogs

**DOI:** 10.3389/fvets.2023.1198134

**Published:** 2023-07-13

**Authors:** Julia Luca, Samantha McCarthy, Thomas Parmentier, Michal Hazenfratz, Alex Zur Linden, Luis Gaitero, Fiona M. K. James

**Affiliations:** ^1^Department of Clinical Studies, Ontario Veterinary College, University of Guelph, Guelph, ON, Canada; ^2^Medical Science, Canadian Academy of Osteopathy, Hamilton, ON, Canada; ^3^Département de sciences cliniques, Faculté de médecine vétérinaire, Université de Montréal, Saint-Hyacinthe, QC, Canada; ^4^Koret School of Veterinary Medicine, The Hebrew University of Jerusalem, Rehovot, Israel

**Keywords:** canine, electroencephalography, epilepsy, survey, EEG technique

## Abstract

**Background:**

Canine epilepsy is a chronic common neurologic condition where seizures may be underreported. Electroencephalography (EEG) is the patient-side test providing an objective diagnostic criterion for seizures and epilepsy. Despite this, EEG is thought to be rarely used in veterinary neurology.

**Objectives:**

This survey study aims to better understand the current canine EEG usage and techniques and barriers in veterinary neurology.

**Methods:**

The online Qualtrics link was distributed via listserv to members of the American College of Veterinary Internal Medicine (ACVIM) Neurology Specialty and the European College of Veterinary Neurology (ECVN), reaching at least 517 veterinary neurology specialists and trainees worldwide.

**Results:**

The survey received a 35% response rate, for a total of 180 participant responses. Fewer than 50% of veterinary neurologists are currently performing EEG and it is performed infrequently. The most common indication was to determine a discrete event diagnosis. Other reasons included monitoring treatment, determining brain death, identifying the type of seizure or epilepsy, localizing foci, sleep disorders, for research purposes, and post-op brain surgery monitorization. Most respondents interpreted their own EEGs. Clinical barriers to the performance of EEG in dogs were mainly equipment availability, insufficient cases, and financial costs to clients.

**Conclusion:**

This survey provides an update on EEG usage and techniques for dogs, identifying commonalities of technique and areas for development as a potential basis for harmonization of canine EEG techniques. A validated and standardized canine EEG protocol is hoped to improve the diagnosis and treatment of canine epilepsy.

## Introduction

Epilepsy is the most common neurological condition in dogs affecting 0.6–0.75% of dogs (1–3). Diagnosis and treatment may be limited in veterinary neurology since diagnostic confirmation is based on subjective criteria such as description of episodes, viewing of episodes, physical and neurologic examinations, as well as unremarkable advanced tests like magnetic resonance imaging (4). None of these provide objective confirmation of seizure events, nor does the caregiver’s history. The latter leads to an underreporting of seizure frequency in dogs as episodes may be missed, particularly while the caregiver is away (5).

Electroencephalography (EEG) is a test providing an objective diagnosis of seizures. While brain function can be measured and assessed using multiple methods, such as positron emission tomography (PET), single photon emission computed tomography (SPECT), functional magnetic resonance imaging (fMRI), and magnetoencephalography (MEG), none of these methods measure brain function in real time at the bedside (6). For this reason, EEG provides a standard for seizure and epilepsy diagnosis (4). The EEG confirmation of seizure (ictal) or interictal activity thus raises the confidence in a diagnosis of canine idiopathic epilepsy to the highest tier, Tier III (4). EEG can differentiate an epilepsy diagnosis from other conditions including episodic or transient paroxysmal disorders (7), behavioral and movement disorders (8–11), or a coma or nonconvulsive seizures (12–17). Despite it being the gold standard test, EEG is thought to be rarely used in veterinary neurology due to various barriers, e.g., labour requirements or cost-effectiveness.

In veterinary medicine, there are not yet standards for EEG usage and technique as there are in human epileptology, making it difficult to compare EEG recordings between dogs, electroencephalographers, and clinics. The last survey examining veterinary EEG usage and technique was conducted over 34 years ago (18). The survey was mailed to 34 neurologists in the United States and Canada, out of which 19 completed and returned the survey (56% response rate). The survey examined questions such as if EEG was being performed, on which species, what type of electrodes were used, what electrode resistance was being used, montage, sensitivity, frequency settings, number of channels used, any other simultaneous recordings (i.e., EKG, respiration, eye movement), usage of photic stimulation, seizure activating procedures, and chemical restraints. At the time, 17/19 respondents reported using EEG in dogs and cats. The most used electrode placement protocol was by Redding and Knecht (1984) (19) using 5 electrodes, (F3, F4, Cz, O1, O2, RF). In the 20th century, EEG machines recorded deflections of a pen on reams of paper (20). Given intervening technological advances and that the survey was completed by such a small group, there is a need to update knowledge of current veterinary EEG practices considering advances in EEG techniques.

The questions arise as to how commonly and by what protocols EEG is currently performed in veterinary neurology. Amongst neurologists, the sense is that EEG is not a commonly performed technique. With a focus on EEG use in dogs, therefore, the hypothesis was that a low proportion of veterinary neurologists use EEG clinically (< 50% of respondents). Further, it was expected to find that the EEG technique has high variability, with the penetrance of any one protocol being less than 20% of those recording EEG routinely amongst respondents. In order to update veterinary EEG literature, the objectives of this study were to understand the current (1) canine EEG usage and its barriers, (2) techniques in veterinary EEG, and (3) the approaches to EEG review.

## Methods

This survey study was approved by the University of Guelph Research Ethics Board (REB# 19–11-004). As internal validation, a focus group of Ontario Veterinary College (OVC) clinicians tested and approved the survey before it was distributed. The survey contains 27 questions in total grouped into three themes. Theme One questions regarding usage and its barriers asked about frequency of EEG use, barriers encountered by both clinicians and pet owners. Theme Two questions asked about equipment type, electrode layout, and typical procedures. Finally theme Three asked about typical approaches to EEG review. See Supplementary Datasheet 1 for all survey questions. Survey questions had several different formats including multiple-choice, yes/no options, slider, and free text. Survey questions were presented as a 20-min online Qualtrics survey. This online Qualtrics link was distributed to members of the veterinary neurology specialist community world-wide via professional listservs including members of the American College of Veterinary Internal Medicine (ACVIM) Neurology Specialty and the European College of Veterinary Neurology (ECVN), reaching at least 517 veterinary neurology specialists and trainees worldwide (estimate provided by listserv moderator for January 2021, by private communication). The survey was available for a total of 5 weeks from November 30, 2021, to January 8, 2022. The surveys were completed anonymously, therefore participants were not able to withdraw their data once they completed and submitted the Qualtrics survey. Two authors (JL, FJ) reviewed the responses and for questions with free-text answers grouped them according to commonalities. Simple descriptive statistics were performed on the responses. Discrete data were tested for normality using the Kolmogorov–Smirnov Test with *p* = 0.05. Mean and standard deviation (SD) were reported for normally distributed data, whereas median and interquartile range (IQR) were reported otherwise. For certain questions (Q18 and Q20), the Qualtrics ‘slider’ question format summarizes the continuous variable output as minimum, maximum, mean, SD, variance, and count, which were reported.

## Results

### Canine EEG usage and its barriers (questions 1–8, Q1-Q8):

**Figure 1:**
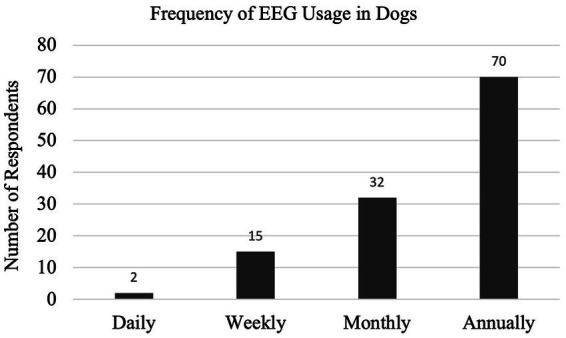
(Q6): Frequency of EEG use for survey respondents.

**Figure 2:**
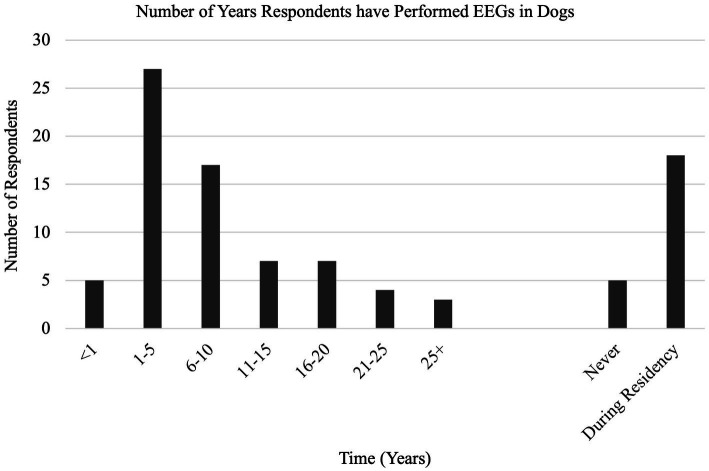
(Q4): Years of experience in performing EEG.

With a 35% response rate, a total of 180 participant responses were recorded. Not all questions were answered by all participants. EEG has been performed at some point by 126/169 (75%) respondents, with 54/123 (44%) respondents performing EEG at the time of survey (Q1, Q2). EEG is most used on an annual basis (70/119, 59%), seen in Figure 1 with the relative frequency at which EEG was performed at the time of the survey (Q6). Indications for performing an EEG are listed in Table 1 (Q3). Figure 2 shows the duration of time in years that respondents have performed EEG (Q4) with the majority being 1–5 years (range 0–49 years). Questions 5, 7, and 8 explored barriers to the uptake of EEG capturing free text answers. The commonalities identified in the answers are summarized in Table 2 along with the frequency of their occurrence. Lack of equipment prevented respondents from performing EEG, whereas number of cases needing EEG affected frequency. Cost to client was frequently cited as affecting client compliance (Table 2).

**Table 1:** (Q3): Indications for performing EEG in dogs.

**Categories**	**Examples of Free Text Response**	**Number of Answers**
Discrete Event Diagnosis (I.e Is it a seizure?)	-Behavior (obsessive compulsive) -Movement disorders (paroxysmal dyskinesia, narcolepsy, syncope without cardiac abnormalities)	76 (63%)
Continuous state diagnosis (I.e Is it still having a seizure?)	- Non-convulsive status, status epilepticus, head trauma, other encephalopathy/intoxication, CCD -Coma	41 (35%)
Drug Monitoring Treatment (I.e Are these drugs working?)	-Drug resistant -Seizure under anesthetic	31 (26%)
Do not perform	Do not perform	20 (17%)
Brain Death	Brain Death	14 (12%)
Type of Seizure/Epilepsy	Focal/absence seizure (5)	11 (9%)
Localizing foci	Localizing foci	4 (3%)
Sleep Disorder	NA	2 (2%)
Research/Academia	NA	4 (3%)
Post-op Brain Surgery Monitoring	NA	1 (1%)
TOTAL		119

**Table 2:** (Q5, 7, 8): Barriers affecting EEG performance in dogs from a veterinarian and owner’s perspective.

**Barrier Factors**	**DVM Stopped Performing (Q5)**	**Affecting DVM frequency (Q7)**	**Affecting Client’s Compliance (Q8)**
Lack of Equipment	38 (37%)	20 (16%)	7 (7.5%)
Lack of Training/Experience	20 (19%)	22 (17%)	NA
Financial Cost	12 (12%)	8 (6%)	26 (28%) *Cost to client
Limited Diagnostic Value	15 (14%)	20 (16%)	1 (1%)
Other Barriers	18 (18%)	55 (43%)	NA
	Lack of staff = 4 (4%)	Lack of staff = 9 (7%)	
	Not enough cases needed for EEG = 3 (3%)	Based on # of cases needing EEG = 26 (21%)	
	Lack of time (use & interpretation) = 11 (11%)	Lack of time = 20 (16%)	
Client Com pliance	Lack of client cooperation = 1 (1%)	Lack of client cooperation = 2 (2%)	Total = 22 (24%) Uncooperative dog = 4 (4%)
			Anesthesia or sedation needed = 9 (10%)
			Far drive to specialist = 2 (2%)
			Hospitalization needed = 7 (8%)
**Total Responses**	104	127	93

### Techniques in veterinary EEG (Q9-Q23):

A wired EEG machine was used by 97/132 (74%) respondents, the remaining used wireless machines (Q9). Video was not used while recording in 60/119 (50%; Q10). Where video was used, 40% (48/119) of respondents recorded video synchronized with the EEG software while the remaining 9% (11/119) recorded video with a separate system, for example, with a GoPro. Subdermal wire electrodes were the most used 88/134 (66%), followed by steel needle electrodes 31/134 (23%), and skin surface electrodes 15/134 (11%; Q11). The types of skin surface electrodes (15; Q12) were reported to be metal (6), disposable cup electrodes (1), patch (3), CCX chloride electrodes (2), silver coated plastic electrodes with T20 paste (1), and “the ones humans have” (2).

Ninety free text responses were submitted for the number of electrodes in the electrode array (Q13). Between 6 to 32 electrodes are being used including ground and reference (median = 12 electrodes, IQR = 6 electrodes; Q13). To explore the electrode array, four images (maps with electrode nomenclature) were presented for selection ( 7, 20, 21, 22) or respondents could upload a map that they use (Table 3; Q14, 15, 16). The Holliday and Williams electrode array map was most frequently selected (44/132, 33%), followed by James et al. (31/132, 24%), Tepper and Shores (26/132, 20%) and lastly Pellegrino and Sica (24/132, 18%). The most uploaded image was taken from Wrzosek (4/9 uploads, 44%) (23). Integrity of electrode placement was typically confirmed via visual inspection of tracings (45/172, 26%), or electrodes (45/172, 36%). Software measures of impedance were also used: <10 kΩ (35/172, 20%), versus <5 kΩ (27/172, 16%) or < 20 kΩ (6/172, 4%). Integrity of electrode placement was not checked by 14/172 (8%) respondents (Q17).

**Table 3:** (Q14) Electrode locations maps commonly used for electrode placement on a dog’s scalp.

**Electrode Location Maps**	**Frequency of Use**	**Number of Electrodes (including ground and reference)**
Holliday TA, Williams DC. Clinical Electroencephalography in Dogs. Vet Neurol Neurosurg J. 1999;1(1):1–38.	44/132 (33%)	15 electrodes
James FMK, Cortez M, Monteith G, et al. Diagnostic utility of wireless video-electroencephalography in unsedated dogs. J Vet Intern. 2017;31(5):1469-1476.	31/132 (24%)	15 electrodes
Tepper L, Shores A. Electroencephalographic recordings in the canine: effects of low dose medetomidine or dexmedetomidine followed by atipamezole. Open J Vet Med. 2014;04:7-13.	26/132 (20%)	6 electrodes
Pellegrino FC, Sica REP. Canine electroencephalographic recording technique: findings in normal and epileptic dogs. Clinical Neurophysiology. 2004;115:477-487.	24/132 (18%)	14 electrodes
Other (respondents could upload image of map on the following Q15.	9/132 (7%)	
Wrzosek MA. Electroencephalography as a diagnostic technique for canine neurological diseases. J Vet Res. 2016 Jun;60:181–7	4/9 (44%)	
Redding R, Knecht C. Atlas of Electroencephalography in the Dog and Cat. (1984)	1/9 (11%)	
Brauer C, Kästner SBR, Schenk HC, Tünsmeyer J, Tipold A. Electroencephalographic recordings in dogs: Prevention of muscle artifacts and evaluation of two activation techniques in healthy individuals. Res Vet Sci. 2011 Apr;90(2):306–11.	1/9 (11%)	
Holliday TA, Williams DC. Advantages of Digital Electroencephalography in Clinical Veterinary Medicine Part 1. Vet Neurol Neurosurg J. 2001;3(1):11.	1/9 (11%)	
Could not tell based on image submitted	2/9 (22%)	

Restraint protocols during instrumentation were ranked as a proportion of cases that the protocol was used for (with a minimum of 0 and a maximum of 100% for each protocol option). Out of 99 responses the mean proportion of use of sedation was 45% (SD = 36, variance = 1,282), ahead of no restraint protocol 30% (97 responses, SD = 34, variance = 1,171), general anesthesia 17% (97 responses, SD = 27, variance = 729), and unspecified other 7% (97 responses, SD = 23, variance = 540; Q18). Awake recording (92 responses, mean 46%, SD = 42, variance = 1783) had a greater proportion of use than sedated (93 responses, mean 35%, SD = 37, variance = 1,376) or anesthetized (93 responses, mean 12%, SD = 25, variance = 620) recordings (with a minimum of 0 and a maximum of 100% for each protocol option; Q20). Alpha-2 agonists were the most used drugs during both instrumentation (46/100, 46%; Q19) and recording (25/64, 39%; Q21). The other drugs listed in the responses to both questions were used less frequently and included propofol, butorphanol, trazadone, phenobarbitone, isoflurane, acepromazine, benzodiazepines. Rocuronium and ketamine were only reported once.

**Figure 3:**
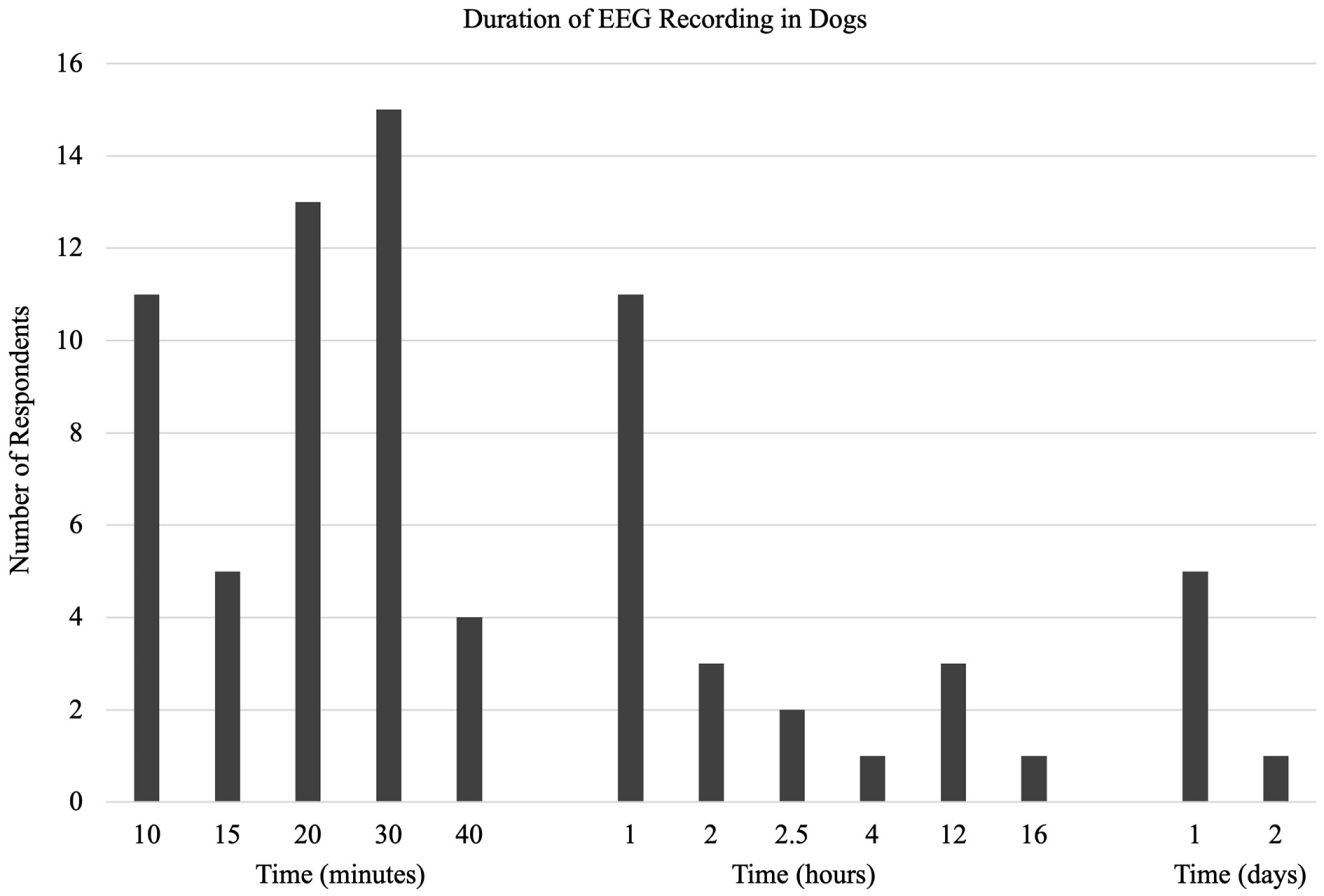
(Q23): EEG recording duration.

Thirty-nine percent (37/94) of respondents did not use anything while fixing electrodes in place. The remaining 61% (57/94) described various methods: adhesive/tape - 30/94, 32%; bandage - 18/94, 19%; shaving - 9/94, 10% (Q22). A typical EEG recording ranged from 10 to 2,880 min (48 h) (median = 30 min, IQR = 40 min; Q23) (Figure 3).

### Approaches to EEG review (Q24-Q27):

In asking whether respondents interpreted their own EEGs, 60% (56/93) selected ‘yes’, 28% (26/93) selected ‘sometimes’, and 12% (11/93) selected ‘no’ (Q24). A follow-up question asked who, other than the respondent, interpreted EEG (Q25). Sixty-three percent (46/73 responses), indicated that they may also consult a colleague, supervisor, friend, or expert for help with interpretation (Q25). As part of EEG interpretation, software algorithms were used by a minority (11/92, 12%; Q26). Respondents reported using the following software: Persyst (3), Polaris, Cadwell Arc Essentia, NicoletOne, NeuroGuide, and iEEG. Three respondents reported using software but did not provide the manufacturer. Both bipolar and reference montages were used for visual review by a majority (49/92, 53%; Q27). The remaining respondents used either bipolar (15/92, 16%), referential (18/92, 20%), or were unsure (10/92, 11%; Q27).

## Discussion

This survey provides an update on EEG usage and techniques for dogs that have evolved since the last survey, over 30 years ago (18). The number of responses (180/517, 35%) represents significant engagement from the veterinary neurology world with a response rate similar to that reported for physician specialist response rates for web-based surveys (24). This support strengthens our conclusions about canine EEG usage and its barriers, techniques in veterinary EEG, and approaches to reviewing EEGs.

### Canine EEG usage and its barriers (Q1-Q8)

Even though most respondents have at one point used EEG in dogs (75%), active usage is lower (44%). This supports the hypothesis that fewer than 50% of veterinary neurologists perform EEGs in practice. The low active usage raises the question whether EEG is performed more frequently during the residency training period. The survey examined barriers to the performance of EEGs in dogs. The most common barrier was lack of available EEG equipment (37%), but insufficient cases also decreased the frequency of EEGs (Table 2). Considering the lower active usage of EEG, a further question is whether EEG units are more likely to be found at centres with residency training programs. A deeper exploration of the availability of EEG units and barriers to their acquisition should be the next step if we are to see more frequent use amongst veterinary neurologists.

Another considerable barrier for veterinary neurologists was lack of training and experience in the procedure itself and its interpretation (19%). This can be addressed by providing advanced continuing education, as well as adjusting learning outcomes within the residency training process. Labour and cost-effectiveness were also factors as respondents were concerned about financial costs associated with EEGs to both the clinic and the pet owners, limited diagnostic value, not enough support staff, and time constraints (Table 2). These factors will likely improve as the body of knowledge advances. As cost effectiveness improves, better client compliance would be expected for consent to an EEG, as the most common barrier was cost (28%, Table 2). Notably the lack of equipment, caseload, and cost are not independent variables. These barriers identify areas for future research and development.

When it is used, EEG is performed annually or less frequently – this cannot be specified due to the limitation of the question format. The most common indication is to determine a discrete event diagnosis – is the dog truly having a seizure or could it be a behavioral or movement disorder? This common indication correlates with the published consensus proposal for diagnosing small animal epilepsy (4), establishing the Tier III confidence level for the diagnosis of idiopathic epilepsy. This indicates broad support for the clinical guidelines of the consensus proposal. Other indications included monitoring treatment, determining brain death, identifying the type of seizure or epilepsy, localizing foci, sleep disorders, for research purposes, and post-op brain surgery monitoring (Table 1) consistent with its use in people (25).

### Techniques in veterinary EEG (Q9-Q23)

The survey findings differed from the predicted high variability in EEG technique. There was less variability than expected in EEG techniques and protocols, with the penetrance of many protocols exceeding 20%. Most respondents reported using a wired EEG machine (74%), versus a wireless EEG machine, perhaps reflecting the age and cost of EEG units in use. Wireless machines are a newer technology, particularly those incorporating synchronized video. Video synchronized recording improves the diagnostic utility for people and dogs (7, 26). The survey found that half of clinicians use video with their EEG recording (50%). If the hypothesis regarding the age of existing EEG units is true, repeating this survey in a few years would demonstrate increased wireless video EEG use.

The most used electrodes are the subdermal wire electrodes (66%), followed by steel needle electrodes (23%) and then skin surface electrodes (11%). This difference from the previously reported use of steel needle and skin surface electrodes only highlights technological advances (18). Subdermal wire electrodes, first described in 2005, have the benefits of low maintenance and durability for longer recordings, as well as advanced imaging compatibility (27, 28). While this survey did not investigate the reasons for the uptake of subdermal wire electrodes, we propose that their popularity is due to their low maintenance requirements.

There was a large range in the number of electrodes used, anywhere between 6 to 32 including ground and reference, with an average of 12 electrodes per recording (mean = 12, median = 12, mode = 12). The most popular electrode array out of the 8 maps reported in our survey was the Holliday and Williams 15-electrode array map which was used by only 33% of respondents (20). This low majority explains the variation seen in the number of electrodes. The previous survey reported the Redding and Knecht 5-electrode array map (1984) as the most common (19). This interesting shift may represent a generational change over the intervening 30 years. Of the 8 maps identified in our survey, the Holliday and Williams map is the oldest (1999) and therefore might be expected to have the greatest penetrance (20). This data will be useful to validate, standardize or harmonize electrode placement arrays. There do appear to be commonalities to these maps which could be used for future harmonization.

The survey identified that quality control is an area for future improvement. The majority of respondents (52%) use a visual inspection of the electrodes and tracings. Only 40% measured impedance. Impedance is a quality measure of the connection between the electrode and scalp (29). Although optimal impedance thresholds have yet to be determined in veterinary EEG, considering previous investigations (30) and the standards in people (22) suggests a threshold of 15 kΩ is reasonable.

In the last survey, there was no distinction made between chemical restraint for instrumentation or recording periods (18). At the time, 6/9 (67%), used no chemical restraint. As it may be more practical to instrument a dog with chemical restraint, the current survey separated the two periods. Despite separating these two periods, the current survey found considerable variation in restraint approaches. Amongst our respondents, sedation is often used for electrode placement, conversely, recordings are often performed without sedation or anesthesia. The higher frequency of both awake recordings and wired EEG units suggests that some form of physical restraint is used, e.g., confinement in a crate or run in the clinic. With the ascendence of wireless EEG technology, the percentage of awake recordings will be expected to increase as it permits the dogs to behave freely.

Of the pharmaceuticals that were reported in instrumentation and recording, alpha-2 agonists dominate compared to the phenothiazine class 30 years ago. The need to understand the effects of pharmaceutical restraint is visible in recent explorations of the topic (22). The class of pharmaceuticals plays into the indications for the EEG, for example, determining the epileptic or non-epileptic nature of paroxysmal episodes and whether these episodes might be abolished by chemical restraint. Phenothiazines were also listed amongst activation techniques in the 1988 survey. Activation techniques were not investigated in the current survey due to the primary focus on EEG usage and its barriers. Recent discussions of activation techniques, like intermittent photic stimulation and hyperventilation, suggest that a more focused survey and research are required (31, 32).

The significant variation in the approach to fixing electrodes in place indicates an area of need. That a large proportion (44%) of EEGs are done without bandaging of some sort may affect the duration and quality of recordings. This was recognized by the one respondent whose technique included “prayer.” That the recording time median and mode were 30 min, but the mean was 3.5 h with a range indicating significant variability, suggested that more work is required to identify the most effective recording period (33). Despite the commonalities in technique, there were some areas that remain open for improvement: number of electrodes, placement map, quality control, and fixation methods. Other technological developments since the previous survey, wireless EEG unit and video recording, may have different indications for use than the standard wired EEG machine. Detection of technology-specific indications was beyond the scope of this survey but would be an interesting area for future research.

### Approaches to EEG review (Q24-Q27)

The current and previous surveys differed with respect to EEG review approaches due to technological advances. The old EEG recordings could not be manipulated post-hoc, which was why the previous survey investigated settings like sensitivity and filters that needed to be adjusted at the time of recording. Nowadays, EEG review software allows adjustments of filters, sensitivity and re-montaging during post-collection review, while some programs even offer automated detection algorithms. In this environment, a high proportion of respondents interpret their own EEG recording without the help software algorithms (60%). Visual inspection rather than software algorithms is the predominant mode for interpretation (87%) likely recognizing that these seizure or spike detection algorithms have yet to be validated for dogs. For visual interpretation, both bipolar and reference montages were used by most respondents (53%) as opposed to reliance on a single montage only, which was a limitation of the pen-and-paper EEG machines. The risk of relying on a single montage for EEG review is an incomplete reconstruction of the three-dimensional cortical potential and is reduced by digital re-montaging post-hoc ( 34). Furthermore, there is a willingness to seek assistance with interpretation from a more experienced colleague, supervisor, or expert (63%), supporting mentorship and collegiality. It is encouraging to find high levels of self-confidence and collaboration regarding review and interpretation.

## Limitations

Similar to the previous survey, this study suffers all the limitations associated with a survey-based design, including respondents being a subset of the target population, incomplete survey responses, response errors, and recall bias. The change in technology limited comparison between the two surveys, meaning that there were slightly different focuses. Administering the survey online and advertising through professional fora extended the survey penetration to the largest audience. Despite 180 respondents, not all questions were answered by all participants. Making the responses anonymous encouraged respondents to provide accurate, honest answers, or even answers that may have presented themselves unfavorably. The converse was that the anonymous responses meant that free text responses could not be linked to earlier responses, e.g., occasional responses reading “see previous answer” (Q14 and Q15), nor could we inquire about career length to normalize timing responses (Q4). The former issue resulted in an unanticipated overlap of results between questions 14 and 15 rendering question 15 less useful, despite initial survey validation. Further, questions 18 and 20 suffered technical glitches with the large response population despite internal preliminary validation; requesting proportions resulted in considerable variation in the results as seen by the large standard deviations and variances. Hindsight also identified at least one question (Q6) where a forced choice limited answers. In retrospect, a larger initial focus group would have identified these issues in the collation of results. To control for recall bias, the survey included images, for example, the electrode map, as well as opportunities for respondents to upload their own images.

## Conclusion

EEG techniques for dogs have evolved over the last 30 years. Fewer than 50% of veterinary neurologists are currently performing EEG and it is performed infrequently. Clinical barriers to the performance of EEG in dogs were mainly equipment availability, insufficient cases, and financial costs to clients. These factors are likely interrelated. Clarity of indications and educational support would build confidence in the use of this diagnostic technique. This survey has identified commonalities of technique and several areas for development. These findings will form the basis for harmonization of canine EEG techniques, thus improving its reliability as a diagnostic test. A validated and standardized canine EEG protocol is hoped to improve the diagnosis and treatment of canine epilepsy. Given the functional similarities between human and dog EEG and epilepsy, basic studies of this nature will support significant advancements in canine epilepsy and EEG with translational implications.

## Data availability statement

The original contributions presented in the study are included in the article/Supplementary material. Further inquiries can be directed to the corresponding author.

## Author contributions

JL, SM, TP, MH, AZ, LG, and FJ: conception, testing, and design. JL and FJ: acquisition and analysis of the data and drafting of the article. JL, SM, TP, MH, AZ, LG, and FJ: revising the article for intellectual content and final approval of completed article.

## Funding

We acknowledge the support of the Natural Sciences and Engineering Research Council of Canada (NSERC), [funding reference number RGPIN-2021-02606].

Cette recherche a été financée par le Conseil de recherches en sciences naturelles et en génie du Canada (CRSNG), [numéro de référence RGPIN-2021-02606].

## Conflict of interest

The authors declare that the research was conducted in the absence of any commercial or financial relationships that could be construed as a potential conflict of interest.

## Publisher’s note

All claims expressed in this article are solely those of the authors and do not necessarily represent those of their affiliated organizations, or those of the publisher, the editors and the reviewers. Any product that may be evaluated in this article, or claim that may be made by its manufacturer, is not guaranteed or endorsed by the publisher.
