# Supplementary material for: Survey of electroencephalography usage and techniques for dogs
Source: Front Vet Sci. 2023 Jul 13;10:1198134. doi: 10.3389/fvets.2023.1198134 (PMC10374286; doi:10.3389/fvets.2023.1198134)
Supplement: Supplementary file 1 [file Data_Sheet_1.pdf]

Introduction

By clicking on the “I AGREE” button below, I acknowledge that I have read the information presented in the information letter about a study being conducted by Julia Luca, MSc., under the supervision of Dr. Fiona James (Ontario Veterinary College, University of Guelph). I understand that I may withdraw from the study at any time up until submission of this survey if I choose to do so, and I agree to participate in this study.

- I AGREE. Take me to the survey
- I DO NOT AGREE. I will not participate right now

Intro Question

1. Have you ever performed EEG in dogs?

- Yes
- No

2. Do you currently perform EEG in dogs?

- Yes
- No

3. For which indications do you perform EEG in dogs?

4. How long have you performed EEG in dogs?

5. If you do not perform EEG in dogs now, why did you stop? (please indicate N/A if still performing EEG in dogs)

6. How often did you/do you use EEG in dogs?

- Daily
- Weekly
- Monthly
- Annually

7. What factors affect your frequency of performing EEG in dogs?

8. What factors affect your client's willingness to perform EEG in dogs?

Technical Protocol

9. Do you use a wired machine or wireless machine when performing EEG in dogs? (select all that apply)

- [ ] Wired
- [ ] Wireless

10. Do you record EEG with video in dogs? (select all that apply)

- [ ] Yes – synchronized video (e.g. within EEG software)
- [ ] Yes – separate video (e.g. with GoPro)
- [ ] No

11. Which electrode types do you use when performing EEG in dogs? (select all that apply)

- [ ] Subdermal wire electrodes
- [ ] Steel needle electrodes
- [ ] Skin surface electrodes

12. If you use skin surface electrodes when performing EEG in dogs, please elaborate: ie. metal, patch, other (please indicate N/A if not applicable)

13. How many electrodes do you use when performing EEG in dogs? (including ground and reference electrodes)

14. Which electrode locations map do you use on the scalp? (select all maps that apply)

- [ ] Holliday TA, Williams C. Clinical electroencephalography in dogs. Website for Veterinary Neurology and Neurosurgery electronic journal, http://www.neurovet.org.

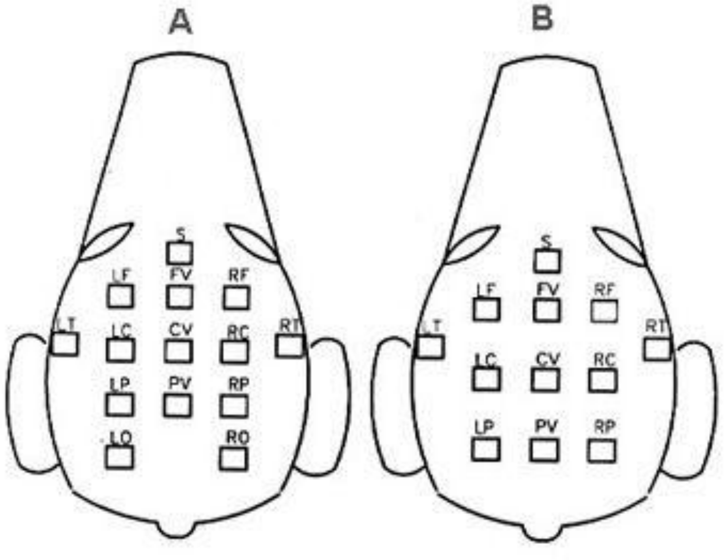

- [ ] Tepper L, Shores A. Electroencephalographic recordings in the canine: effects of low dose medetomidine or dexmedetomidine followed by atipamezole. Open J Vet Med. 2014;04:7–13.

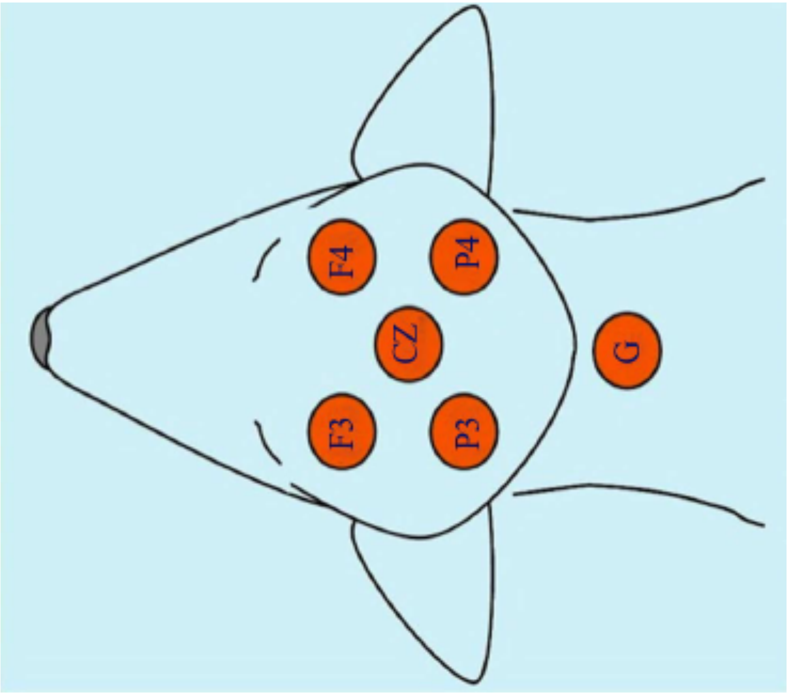

- [ ] Pellegrino FC, Sica REP. Canine electroencephalographic recording technique: findings in normal and epileptic dogs. Clinical Neurophysiology. 2004;115:477-487.

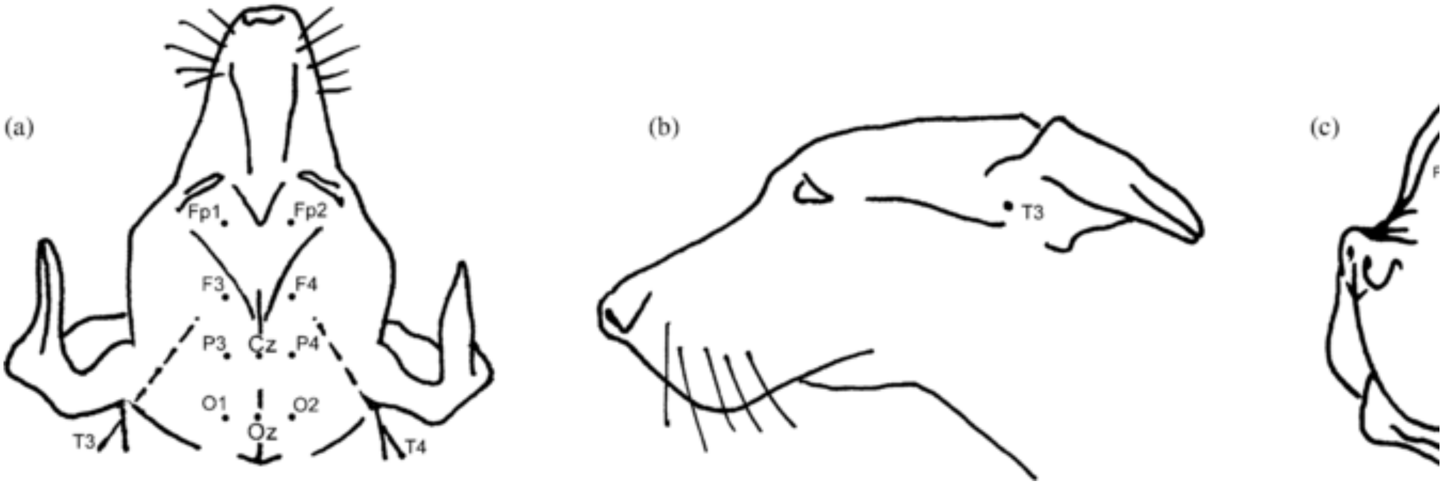

- [ ] James FMK, Cortez M, Monteith G, et al. Diagnostic utility of wireless video-electroencephalography in unsedated dogs. J Vet Intern. 2017;31(5):1469-1476.

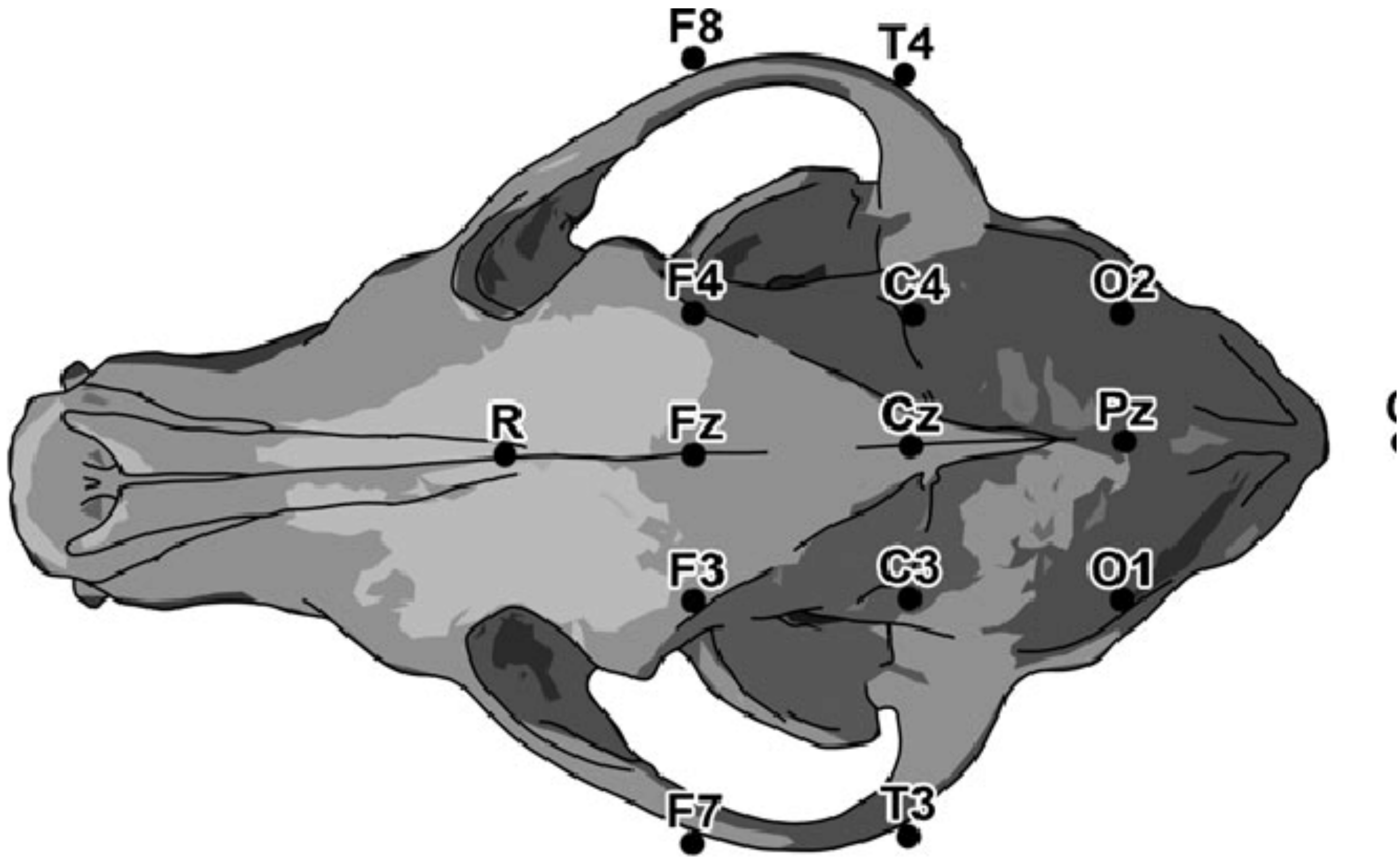

☐ Other (please upload image on following questions)

15. Describe the nomenclature you use for electrode placement when performing EEG in dogs (example: Fp1/Fp2, Cz, RO or LO; option to upload image next question)

16. If available, please upload an image of your electrode placement protocol when performing EEG in dogs

17. How do you confirm integrity of electrode placement?

- [ ] Software measures impedance <5 kOhms
- [ ] Software measures impedance <10 kOhms
- [ ] Software measures impedance <20 kOhms
- [ ] Visual inspection of electrodes
- [ ] Visual inspection of tracings
- [ ] We don't check

18. Which restraint protocols do you use **during placement** when performing EEG in dogs? (indicate proportions, equaling a total of 100%)

Percentage of cases

	0	10	20	30	40	50	60	70	80	90	100
Sedation											
General anesthetic											
None											
Other											

Total: **0**

19. If using drugs **during instrumentation** when performing EEG in dogs, please list drugs used (indicate N/A if not applicable)

20. Which restraint protocols do you use **during recording** when performing EEG in dogs? (indicate proportions, equaling a total of 100%)

Percentage of cases

0 10 20 30 40 50 60 70 80 90 100

Sedation

General anesthetic

None

Other

Total: **0**

21. If using drugs **during recording** when performing EEG in dogs, please list drugs used (indicate N/A if not applicable)

22. Please describe your bandage protocol for fixing electrodes when performing EEG in dogs (example: shaved head, adhesive, etc.)

23. How long is a typical recording when performing EEG in dogs?

Review Techniques

24. Do/did you interpret your own EEGs when performing EEG in dogs?

- Yes
- Sometimes
- No

25. If you indicated that someone other than yourself interprets any EEG results when performing EEG in dogs, please describe who and explain why

26. As part of interpretation when performing EEG in dogs, do/did you utilize software algorithms? (select all that apply)

- ☐ No
- ☐ Yes (please list software)

Montage

27. Which montage(s) did/do you use when reviewing EEGs in dogs?

- ○ Bipolar
- ○ Reference
- ○ Both bipolar and reference
- ○ Other (please explain)

Survey Powered By Qualtrics
